# Differential expression of micrornas in porcine parvovirus infected porcine cell line

**DOI:** 10.1186/s12985-015-0359-4

**Published:** 2015-08-20

**Authors:** Xinqiong Li, Ling Zhu, Xiao Liu, Xiangang Sun, Yuanchen Zhou, Qiaoli Lang, Ping Li, Yuhan Cai, Xiaogai Qiao, Zhiwen Xu

**Affiliations:** Animal Biotechnology Center, College of Veterinary Medicine, Sichuan Agricultural University, Wenjiang, Chengdu, China; Key Laboratory of Animal Disease and Human Health, College of Veterinary Medicine, Sichuan Agricultural University, Wenjiang, Chengdu, China

## Abstract

**Background:**

Porcine parvovirus (PPV), a member of the Parvoviridae family, causes great economic loss in the swine industry worldwide. MicroRNAs (miRNAs) are a class of non-protein–coding genes that play many diverse and complex roles in viral infections.

**Finding:**

Aiming to determine the impact of PPV infections on the cellular miRNAome, we used high-throughput sequencing to sequence two miRNA libraries prepared from porcine kidney 15 (PK-15) cells under normal conditions and during PPV infection. There was differential miRNA expression between the uninfected and infected cells: 65 miRNAs were upregulated and 128 miRNAs were downregulated. We detected the expression of miR-10b, miR-20a, miR-19b, miR-181a, miR-146b, miR-18a, and other previously identified immune-related miRNAs. Gene Ontology analysis and KEGG function annotations of the host target genes suggested that the miRNAs are involved in complex cellular pathways, including cellular metabolic processes, immune system processes, and gene expression.

**Conclusions:**

These data suggest that a large group of miRNAs is expressed in PK-15 cells and that some miRNAs were altered in PPV-infected PK-15 cells. A number of microRNAs play an important role in regulating immune-related gene expression. Our findings should help with the development of new control strategies to prevent or treat PPV infections in swine.

## Background

Porcine parvovirus (PPV) is a major cause of reproductive failure in swine (*Sus scrofa*, ssc), where infection is characterized by early embryonic death, stillbirths, fetal death, and delayed return to estrus [1]. Additionally, PPV is associated with porcine postweaning multisystemic wasting syndrome (PMWS) and diarrhea, skin disease, and arthritis in swine [1, 2]. Even though inactivated and attenuated vaccines are widely used, the PPV-associated diseases nevertheless cause serious economic losses to the swine industry worldwide [3]. As virus replication is highly dependent on the host cell, cellular microRNA (miRNA) modification of the complex cellular regulatory networks can greatly influence viral reproduction and pathogenesis. Therefore, determining the consequences of PPV infections on cellular gene regulatory networks is urgent.

miRNAs are involved in post-transcriptional regulation of gene expression in animals, plants, and some DNA viruses. miRNAs act as regulators, inhibiting the expression of specific mRNAs by recognizing partial complementary sites in a targeted mRNA, typically within the 3’ untranslated region (3’UTR). miRNAs perform critical functions in diverse biological processes, including proliferation, apoptosis, and cell differentiation [4]. It has been well established that miRNAs play many complex roles during viral infection [5]. Therefore, an increasing number of researchers have focused on the relationship between viruses and miRNAs.

As far as we know, knowledge on the role of miRNAs in PPV infection is lacking. In this study, we detected the miRNAs expressed in porcine kidney 15 (PK-15) cells following PPV infection using high-throughput sequencing.

## Methods

We used the PPV-SC-L strain, stored at the Key Laboratory of Animal Diseases and Human Health of Sichuan Province, China, in this study. PK-15 cell cultures that were 50 % confluent were infected with PPV at 10 plaque-forming units (PFU) per cell. PK-15 cells inoculated with DMEM were maintained as uninfected control cells. Cells were harvested at 24 h post-infection [6]. The cultures for each group were performed in triplicate. The infected and uninfected cells were mixed separately and used for RNA extraction. Cell viability is not affected during timecourse of infection.

Total RNA from infected PK-15 cells and normal PK-15 cells was extracted using TRIzol (Invitrogen) according to the manufacturer’s instructions. RNA quality was assessed by formaldehyde/agarose gel electrophoresis and was quantified using a ND-1000 NanoDrop Spectrophotometer (Thermo Scientific, Wilmington, MA, USA). Approximately 20 μg total RNA was subjected to Kangcheng Bio-tech inc (Shanghai, China) for Solexa sequencing of miRNAs. The same RNA was used for qRT-PCR.

RT was performed as previously described [6]. Real-time PCR was performed using SYBR Green Real-time qPCR Master Mix (Arraystar, Rockville, MD, USA) on a ViiA 7 Real-Time PCR System (Applied Biosystems, Foster City, CA, USA) according to the manufacturer’s instructions. The amplification conditions were as follows: 95 °C for 10 min, followed by 40 cycles of 95 °C for 10 s and 60 °C for 60 s. Table 1 lists the primers used. All samples were assayed in triplicate. The cycle threshold (Ct) values were analyzed using the 2^-∆∆Ct^ method. The *U6* gene was used as the internal control.

**Table 1:** RT-qPCR primers

Gene	RT primer	
U6	5’CGCTTCACGAATTTGCGTGTCAT3’	
miR-RT Primer	5’GTCGGTGTCGTGGAGTCGTTTGCAATTGCACTGGATTTTTTTTTTTTTTTTTTV3’	
V = A, G, C		
Gene	Forward primer (5’–3’)	Reversed primer (5’–3’)
ssc-miR-10b	TACCCTGTAGAACCGAATTTGT	GTCGGTGTCGTGGAGTCG
ssc-miR-30a-5p	TGTAAACATCCTCGACTGGAAG	GTCGGTGTCGTGGAGTCG
ssc-miR-16	TAGCAGCACGTAAATATTGGC	GTCGGTGTCGTGGAGTCG
ssc-miR-17-5p	CAAAGTGCTTACAGTGCAGGTAG	GTCGGTGTCGTGGAGTCG
ssc-miR-192	CTGACCTATGAATTGACA	GTCGGTGTCGTGGAGTCG
ssc-miR-21	TAGCTTATCAGACTGATGTTGA	GTCGGTGTCGTGGAGTCG
ssc-miR-19b	TGTGCAAATCCATGCAAAAC	GTCGGTGTCGTGGAGTCG
ssc-miR-18a	TAAGGTGCATCTAGTGCAGATA	GTCGGTGTCGTGGAGTCG
ssc-miR-152	TCAGTGCATGACAGAACTTGG	GTCGGTGTCGTGGAGTCG
ssc-miR-novel-chr13_10861	TTCAAGTAACCCAGGATAGGCT	GTCGGTGTCGTGGAGTCG
U6	TCGCTTTGGCAGCACCTAT	AATATGGAACGCTTCGCAAA

MiRanda and TargetScan were used to predict the targets of the differentially expressed miRNAs. Predicted miRNA targets were functionally annotated through the cell component, biological process, and molecular function information supported by GO analysis. GO analysis and KEGG pathway analyses were performed using DAVID (http://david.abcc.ncifcrf.gov/) with default parameters [7].

## Results

**Fig. 1:**
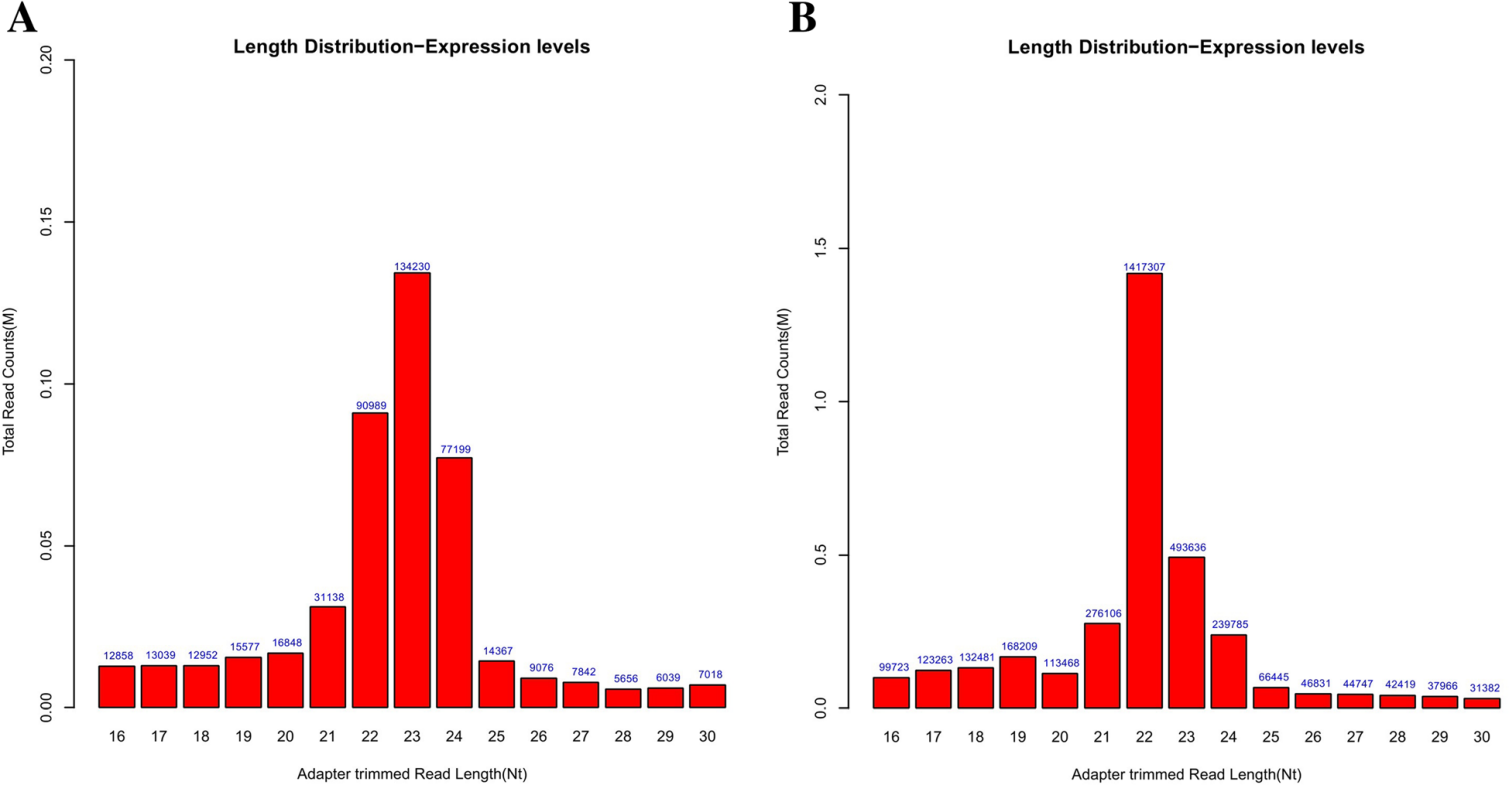
Length distribution of miRNA reads from Solexa sequencing. **a** Adapter-trimmed reads in the infected library; **b** adapter-trimmed reads in the control library

We obtained 3,575,737 and 617,535 high-quality reads from the normal and infected cell samples, respectively, remained for miRNA analysis. The length distribution of the high-quality reads ranged 16–30 nt. Most sequence reads ranged 21–24 nt, which belonged to the typical size range (Fig. 1). We identified 533 and 286 porcine miRNAs in normal PK-15 cells and infected PK-15 cells, respectively. This indicates that the normal cells contained more miRNAs than the infected cells. The change of expression of miRNAs between normal and infected PK-15 cells reflects that miRNAs can play key roles during the viral infection process, where virus can affect cellular miRNAs expression profile on their own benefit. ssc-miR-21 was the most abundantly expressed miRNA, followed by ssc-miR-30a-5p. miRNAs were considered differentially expressed when the fold change (FC) difference between groups was >2 or ≤0.5 and *P* ≤ 0.01, or when a miRNA was not expressed in either the infected or control group. There were 193 differentially expressed miRNAs; 128 were downregulated and 65 were upregulated. The most upregulated and downgulated miRNA were ssc-miR-10b (36-fold) and ssc-miR-18a (0.01-fold) (Table 2).

**Table 2:** Top 50 miRNAs significantly up- or downregulated in PK-15 cells in order of fold change (FC)

Annotation	Normalized read counts		length	type	FC	Number of target genes
	infected	control				
ssc-miR-10b	42,588	1162	22	Up	36.35	738
ssc-miR-192	3769	102	21	Up	33.74	718
ssc-miR-20a	2432	116	22	Up	19.38	1490
ssc-miR-296-3p	195	3	21	Up	15.77	1863
ssc-miR-novel-chr17-18987	195	3	19	Up	15.77	1864
ssc-miR-92b-3p	2215	133	22	Up	15.56	1757
ssc-miR-30a-5p	98,034	6320	22	Up	15.49	1147
ssc-miR-novel-chr12-7961	1886	191	22	Up	9.43	1357
ssc-miR-novel-chr14-13888	582	58	23	Up	8.71	1368
ssc-miR-34a	358	37	22	Up	7.83	1663
ssc-miR-novel-chr16-17559	55	0	22	Up	6.5	1610
ssc-miR-novel-JH11865-1-42	55	0	23	Up	6.5	1727
ssc-miR-17-5p	2868	438	23	Up	6.42	1443
ssc-miR-16	11,873	1891	22	Up	6.25	1763
ssc-miR-22-3p	2267	365	22	Up	6.07	1487
ssc-miR-146b	75	3	21	Up	6.07	1139
ssc-miR-155-5p	426	62	22	Up	6.06	1146
ssc-miR-novel-chr2-20965	52	1	23	Up	5.64	1147
ssc-miR-novel-chrx-40705	758	147	22	Up	4.89	811
ssc-miR-221-3p	758	147	22	Up	4.89	811
ssc-miR-301	114	17	23	Up	4.59	1509
ssc-miR-191	741	156	23	Up	4.52	695
ssc-miR-novel-chr6-31692	46	3	22	Up	4.30	2019
ssc-miR-181a	637	147	24	Up	4.12	1221
ssc-miR-18a	88	9541	22	Down	0.0102	995
ssc-miR-novel-chr9-37990	20	1752	23	Down	0.0170	1512
ssc-miR-novel-chr9-39041	20	1752	23	Down	0.0170	1512
ssc-miR-novel-chr6-30729	13	1317	22	Down	0.0173	1083
ssc-miR-424-5p	33	2182	22	Down	0.0196	1817
ssc-miR-31	55	3118	22	Down	0.0208	1149
ssc-miR-novel-chrX-41190	0	431	21	Down	0.0227	335
ssc-miR-novel-chr11-6750	7	547	18	Down	0.0305	1406
ssc-miR-152	332	9880	21	Down	0.0346	1161
ssc-miR-542-5p	0	277	21	Down	0.0348	732
ssc-miR-499-5p	7	472	21	Down	0.0353	974
ssc-miR-142-3p	0	238	22	Down	0.0403	887
ssc-miR-135	0	235	23	Down	0.0408	1602
ssc-miR-194a	13	541	21	Down	0.0417	842
ssc-miR-361-5p	20	704	22	Down	0.0420	867
ssc-miR-185	59	1621	22	Down	0.0423	2285
ssc-miR-193a-5p	0	201	22	Down	0.0474	1142
ssc-miR-novel-chr5-29676	0	199	23	Down	0.0478	1066
ssc-miR-183	156	3132	23	Down	0.0528	1087
ssc-miR-29c	16	366	22	Down	0.0691	1120
ssc-miR-novel-chr5-29857	42	736	19	Down	0.0697	1711
ssc-miR-29a	267	3939	23	Down	0.0701	1079
ssc-miR-19a	498	6339	23	Down	0.0800	1436
ssc-miR-19b	1161	14,587	23	Down	0.0802	1299
ssc-miR--novel-chr13_10861	169	1483	22	Down	0.1199	857
ssc-miR-21	52,611	382,830	22	Down	0.1374	789

**Fig. 2:**
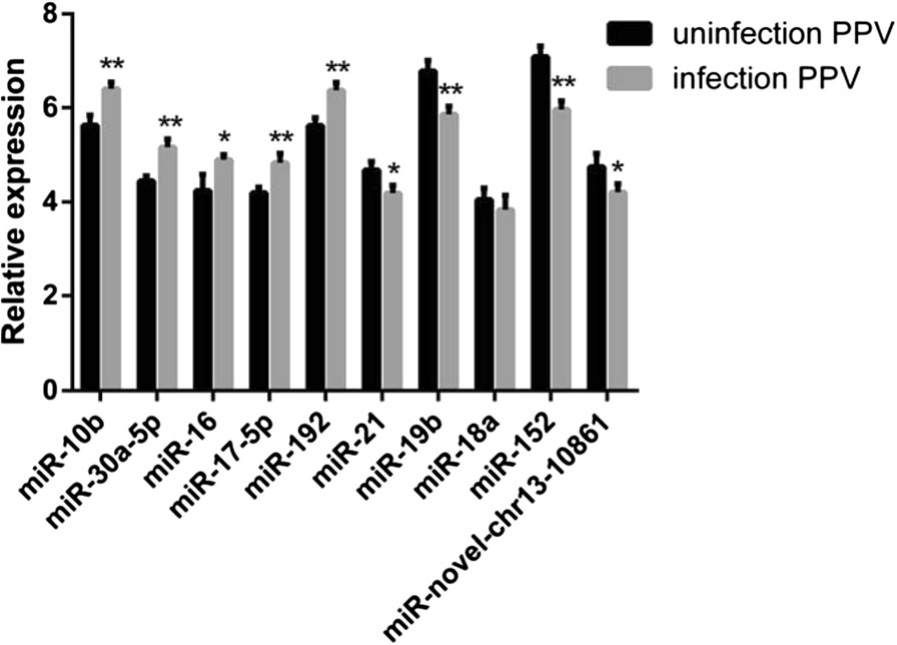
RT-qPCR validation and expression analysis of differentially expressed miRNAs. The relative expression levels are presented as the mean and standard deviation (SD). **P < 0.01, *P < 0.05

We selected 10 miRNAs to confirm the deep sequencing data. The expression levels of ssc-miR-10b, ssc-miR-30a-5p, ssc-miR-16, ssc-miR-17-5p, and ssc-miR-192 in the PPV-infected cells were higher than in the uninfected cells, whereas ssc-miR-21, ssc-miR-19b, ssc-miR-18a, ssc-miR-152, and ssc-miR-novel-chr13_10861 were downregulated compared to the uninfected cells (Fig. 2). The results were consistent with that of the deep sequencing analysis. In addition, reverse transcription–quantitative PCR (RT-qPCR) indicated the reliability of the deep sequencing data.

In our study, a total 3254 target genes were predicted for the 193 differentially expressed miRNAs. We successfully annotated about 2867 target genes through GO analysis. The upregulated biological process–related genes were involved in cellular process, metabolic process and biological regulation. The biological roles of the downregulated genes were cellular process, metabolic process, and biological regulation. GO enrichment analysis determined functional enrichment of upregulated and downregulated genes in cellular process and cell part and binding (Table 3). The target genes were classified according to Kyoto Encyclopedia of Genes and Genomes (KEGG) function annotations, and we identified pathways actively regulated by the miRNAs during PPV infection (Table 4). Some of the target genes were involved in immunity and virus infection.

**Table 3:** GO analysis of swine target genes. The table shows the GO annotation of the upregulated gene (A) and downregulated gene (B) in biological process, cellular component and molecular function. Ten GO terms for each process are listed

GO.ID	Term	Count	*P*-value
Biological process			
GO:0009987	cellular process	1782	1.0102E-05
GO:0008152	metabolic process	1350	2.44953E-27
GO:0065007	biological regulation	1260	0.000424577
GO:0044238	primary metabolic process	1231	5.99319E-26
GO:0044237	cellular metabolic process	1221	1.70495E-28
GO:0050789	regulation of biological process	1192	0.002408788
GO:0050794	regulation of cellular process	1147	0.000216533
GO:0002376	immune system process	273	1.35305E-08
GO:0006955	immune response	163	1.37682E-05
GO:0000165	MAPK cascade	79	3.28195E-05
Cellular Component			
GO:0044464	cell part	1772	1.04304E-42
GO:0005623	cell	1772	1.25735E-42
GO:0005622	intracellular	1589	1.48695E-38
GO:0044424	intracellular part	1512	9.75601E-38
GO:0043226	organelle	1258	3.15768E-22
GO:0043229	intracellular organelle	1255	5.59497E-22
GO:0005737	cytoplasm	1146	1.88382E-25
GO:0043227	membrane-bounded organelle	1131	3.1329E-23
GO:0043231	intracellular membrane-bounded organelle	1129	3.31685E-23
GO:0044444	cytoplasmic part	886	1.59538E-15
Molecular Function			
GO:0005488	binding	1781	7.2806E-35
GO:0005515	protein binding	1406	1.01651E-30
GO:0003824	catalytic activity	791	4.12422E-11
GO:0043167	ion binding	425	2.86598E-07
GO:0043169	cation binding	423	3.71961E-07
GO:0046872	metal ion binding	416	4.07808E-07
GO:0003676	nucleic acid binding	368	0.010086661
GO:0036094	small molecule binding	366	1.33205E-09
GO:0000166	nucleotide binding	341	4.24208E-09
GO:0097159	organic cyclic compound binding	341	4.47117E-09
B			
Biological Process			
GO:0009987	cellular process	1732	0.000468457
GO:0008152	metabolic process	1280	0.000101041
GO:0065007	biological regulation	1226	0.039474247
GO:0044238	primary metabolic process	1179	0.011728824
GO:0044237	cellular metabolic process	1153	0.011728824
GO:0050789	regulation of biological process	1150	0.022891558
GO:0050794	regulation of cellular process	1100	0.023393923
GO:0002376	immune system process	265	3.49438E-08
GO:0006955	immune response	161	7.5883E-06
GO:0022402	cell cycle process	151	0.001985807
Cellular Component			
GO:0044464	cell part	1699	1.61069E-32
GO:0005623	cell	1699	1.89406E-32
GO:0005622	intracellular	1506	1.8551E-26
GO:0044424	intracellular part	1426	4.89384E-25
GO:0043226	organelle	1212	8.24637E-20
GO:0043229	intracellular organelle	1208	2.30659E-19
GO:0043227	membrane-bounded organelle	1089	8.12018E-21
GO:0043231	intracellular membrane-bounded organelle	1088	5.50768E-21
GO:0005737	cytoplasm	1079	5.97213E-18
GO:0044444	cytoplasmic part	836	2.48602E-11
Molecular Function			
GO:0005488	binding	1748	3.63499E-36
GO:0005515	protein binding	1408	1.06202E-38
GO:0003824	catalytic activity	743	5.00132E-07
GO:0043167	ion binding	410	2.18765E-06
GO:0043169	cation binding	408	2.81762E-06
GO:0046872	metal ion binding	400	4.31756E-06
GO:0003676	nucleic acid binding	365	0.004482893
GO:0036094	small molecule binding	335	6.20944E-06
GO:0000166	nucleotide binding	309	2.81002E-05
GO:0097159	organic cyclic compound binding	309	2.91504E-05

**Table. 4:** Target genes of 17 differentially expressed miRNAs involved in immune response pathways

KEGG pathways	Target genes	Differentially expressed microRNAs	FDR
T cell receptor signaling pathway	CTLA4, FYN, IKBKG, NFATC2, NCK1, CD8A, PIK3CG, CDC42, PTPN6, CD4, CD40LG, ICOS, PIK3R5, MAPK14, TNF, MAP3K7	miR-10b, miR-9, miR-30a-5p, miR-17-5p, miR-16, miR-18a, miR-19b, miR-20a, miR-19a, miR-122, miR-146b, miR-55-5p, miR-181a, miR-196b, let-7 g, let-7c	8.89308E-12
Toll-like receptor signaling pathway	CTSK, TLR7, MAP3K7, MAPK14, CXCL9, PIK3CG, NFKB1, CD40, STAT1, IL12B, CD86, IL6	miR-10b, miR-9, miR-30a-5p, miR-17-5p, miR-16, miR-18a, miR-19b, miR-20a, miR-21, miR-19a, miR-122, miR-146b, miR-155-5p, miR-181a, miR-196b, let-7 g, let-7c	1.04578E-07
NF-kappaB signaling pathway	MAP3K7, CXCL12, DDX58, LCK, XIAP, ATM, VCAM1, NFKB1, TNF, CD40LG	miR-10b, miR-9, miR-30a-5p, miR-17-5p, miR-16, miR-18a, miR-19b, miR-20a, miR-19a, miR-122, miR-146b, miR-155-5p, miR-181a, miR-196b	1.18108E-06
RIG-I-like receptor signaling pathway	MAP3K7, MAPK14, DHX58, DDX58, IKBKG, TANK, IKBKB, DDX3X, NFKB1, TNF, IL12B	miR-10b, miR-9, miR-30a-5p, miR-17-5p, miR-16, miR-18a, miR-19b, miR-21, miR-19a, miR-122, miR-146b, miR-155-5p, miR-181a, let-7c	1.70355E-05
Jak-STAT signaling pathway	JAK2, STAT4, STAT5B, JAK3, PIK3CG, PIM1, PTPN6, TYK2, MAPK14, STAT4, STAT1, IL7R, IL12B, IL6, PIK3R5, MYC	miR-9, miR-17-5p, miR-16, miR-18a, miR-19b, miR-20a, miR-21, miR-19a, miR-122, miR-146b, miR-155-5p, miR-181a, miR-196b, let-7 g, let-7c	0.000124339
NOD-like receptor signaling pathway	MAP3K7, MAPK14, IKBKG, IKBKB, NFKB1, TNF, IL6	miR-10b, miR-9, miR-17-5p, miR-16, miR-18a, miR-19b, miR-19a, miR-122, miR-146b, miR-155-5p, miR-181a, let-7 g, let-7c	0.001546381

## Discussion and conclusion

Previous studies have shown that viruses have evolved a wide variety of means for resisting the host immune system [8–10]. Furthermore, miRNAs play important roles in controlling immune regulation, including cellular differentiation and immune response [11–13]. Identifying and probing miRNAs in the immune system is important for understanding their physiological and pathological roles in PPV infection. In this study, we used high-throughput sequencing to identify miRNAs.

Recent studies have provided compelling evidence that cellular miRNAs play an important role in host defense against virus infection [14]. Many immune-related miRNAs have been identified in innate and adaptive immune systems, including the miR-17—92 cluster, miR-221, miR-10, miR-196b, miR-126, miR-155, miR-150; miR-181a, miR-326, miR-142-3p, miR-424, miR-21, miR-106a, miR-223, miR-146; the let-7 family, miR-9, and miR-34 [6]. We found many differentially expressed miRNAs in the normal and PPV-infected PK-15 cells. Among them, let-7 g, miR-17-5p, miR-17-3p, miR-20a, miR-181a, miR-16, miR-146b, miR-10b, and miR-155-5p were upregulated; let-7c, miR-122, miR-18a, miR-19a, miR-19b, miR-196b, miR-21, and miR-9 were downregulated. These data suggest that viral mechanisms can affect host miRNA expression. However, we did not detect differential expression of other previously identified miRNAs (miR-223, miR-150, miR-92a), although miR-10b, miR-20a, miR-30a-5p, miR-34a, miR-17—5p, miR-16, miR-146b, and miR-155-5p expression was significantly different. In contrast, expression of the downregulated immune-related miRNAs was not significantly different, except miR-18a, miR-19b, and miR-21. This suggests that miRNAs play an important role in the coordinated regulation of immune-related gene expression in PK-15 cells in response to PPV infection.

miR-21, which had high read numbers in both normal and PPV-infected cells, was downregulated; it is related to immune response and virus replication [15]. Moreover, it is a negative regulator of toll-like receptor 4 (TLR4) signaling by targeting programmed cell death 4 (PDCD4) [16]. miR-19b and miR-18a expression was downregulated in the infected cells, suggesting that they play a negative role in PPV replication. Although viruses may downregulate host miRNA by suppressing Dicer expression, the mechanism of downregulation remains unclear [17]. Therefore, future studies are necessary for investigating the mechanism of PPV downregulation of cellular miRNA.

miR-10 expression was upregulated in the infected cells. Mitogen-activated protein kinase kinase kinase 7 (*MAP3K7*), considered a target gene of miR-10, regulates the inhibitor of nuclear factor κB/nuclear factor κB (IκB/NF-κB) signaling pathway [18]. In addition, miR-10 controls brain-derived neurotrophic factor (BDNF) levels via the miRNA–mRNA regulatory network [19]. We surmise that a possible function of miR-10 in triggering an antiviral response is targeting the *MAP3K7* and *BDNF* genes. The miR-30 family is involved in various biological and pathological processes. For example, miR-30a may be involved in B cell hyperactivity [20]. We detected miR-10 and miR-30 in this study, suggesting that they are related to the cellular immune response to PPV infection.

GO analysis showed that many of the identified miRNAs found in other studies were predicted to participate in immunity [21]. Many genes, including *MAP3K7*, *IRAK1*, *TLR7*, *CD40*, *TGFBR1*, *RPS6KA3*, *IGF1R*, *CDC37*, *ITGA4*, *CBLB*, *ITGA5*, *IL7*, *ATM*, *DPP8*, *MAPK14*, *CD2*, *WNT2B*, *CAV1*, and *CD96*, are involved in the immune-related programs. KEGG analysis showed that these targeted genes could participate in multiple signaling pathways, including that for retinoic acid–inducible gene-I (RIG-I)-like receptor, TLRs, Janus kinase–signal transducer and activator of transcription (JAK–STAT), and T-cell receptor. Interleukin 10 (IL10) plays an important role in virus infection by inhibiting several proinflammatory cytokines [22]. Let-7 g, let-7c, miR-19b, and miR-16 are involved in immune-related programs and may act through the target gene *IL10*. These results suggest that miRNAs participate in the regulation of piglet immunity. It has been established that miRNAs can target specific genes [23]; in the present study, let-7c, let-7 g, miR-18a, miR-196b, and miR-9 targeted *MAP3K7*, and miR-196b and miR-19b targeted dipeptidyl-peptidase 8 (*DPP8*), suggesting that cellular miRNAs play a key role in regulating gene expression in response to PPV infection. Genes targeted by miRNAs are involved in immune response–associated pathways in human parvovirus B19 infection [24]. We speculate that host miRNAs relate to common immune pathways in response to parvovirus infection.

To our knowledge, this is first study to survey the miRNA expression profiles in PPV-infected PK-15 cells through high-throughput sequencing. A number of miRNAs detected were previously described as immune system regulators. Target analysis confirmed that these miRNAs played an important role in PPV infection. These findings contribute to our understanding of the roles miRNAs play in host–pathogen interactions and help with the development of new control strategies to prevent or treat PPV infections in swine.
